# Food Insecurity and Children’s Mental Health: A Prospective Birth Cohort Study

**DOI:** 10.1371/journal.pone.0052615

**Published:** 2012-12-26

**Authors:** Maria Melchior, Jean-François Chastang, Bruno Falissard, Cédric Galéra, Richard E. Tremblay, Sylvana M. Côté, Michel Boivin

**Affiliations:** 1 INSERM U1018, Centre for research in Epidemiology and Population Health, Epidemiology of occupational and social determinants of health, F-94807, Villejuif, France; 2 Université de Versailles Saint-Quentin, UMRS 1018, France; 3 INSERM U669, Maison de Solenn, Université Paris-Sud and Université Paris-Descartes, 97 Bd du Port Royal, F-75679 Paris, France; 4 Service de Pédopsychiatrie universitaire, Hôpital Charles-Perrens, Université Victor Ségalen Bordeaux 2, Bordeaux, France; 5 International Laboratory for Child and Adolescent Mental Health, Research Group on Children’s Psychosocial Maladjustment, University of Montreal, Montreal, Canada; 6 School of Public Health and Population Science, University College, Dublin, Ireland; 7 Université Laval, Québec, Canada; Wayne State University, United States of America

## Abstract

Food insecurity (which can be defined as inadequate access to sufficient, safe, and nutritious food that meets individuals’ dietary needs) is concurrently associated with children’s psychological difficulties. However, the predictive role of food insecurity with regard to specific types of children’s mental health symptoms has not previously been studied. We used data from the Longitudinal Study of Child Development in Québec, LSCDQ, a representative birth cohort study of children born in the Québec region, in Canada, in 1997–1998 (n = 2120)**.** Family food insecurity was ascertained when children were 1½ and 4½ years old. Children’s mental health symptoms were assessed longitudinally using validated measures of behaviour at ages 4½, 5, 6 and 8 years. Symptom trajectory groups were estimated to identify children with persistently high levels of depression/anxiety (21.0%), aggression (26.2%), and hyperactivity/inattention (6.0%). The prevalence of food insecurity in the study was 5.9%. In sex-adjusted analyses, children from food-insecure families were disproportionately likely to experience persistent symptoms of depression/anxiety (OR: 1.79, 95% CI 1.15–2.79) and hyperactivity/inattention (OR: 3.06, 95% CI 1.68–5.55). After controlling for immigrant status, family structure, maternal age at child’s birth, family income, maternal and paternal education, prenatal tobacco exposure, maternal and paternal depression and negative parenting, only persistent hyperactivity/inattention remained associated with food insecurity (fully adjusted OR: 2.65, 95% CI 1.16–6.06). Family food insecurity predicts high levels of children’s mental health symptoms, particularly hyperactivity/inattention. Addressing food insecurity and associated problems in families could help reduce the burden of mental health problems in children and reduce social inequalities in development.

## Introduction

In industrialized countries, approximately 5–15% of families experience food insecurity, that is insufficient access to “sufficient, safe, and nutritious food that meets individuals’ dietary needs and preferences for an active and healthy life” [1]–[6]. Prior research has shown that food insecurity is associated with poor health and developmental outcomes in children [7]–[13]. In particular, children growing up in families that are food-insecure appear to have high levels of symptoms of anxiety/depression [12], [14]–[16], aggression, and hyperactivity/inattention [5], [12], [17]. This may be due to three mechanisms: 1) food insecurity may be associated with other exposures related to children’s psychological well-being (e.g. low income); 2) food insecurity and children’s mental health may have common causes (e.g. parental psychopathology); 3) food insecurity may independently predict children’s psychological and behavioral well-being [18]; and 4) food insecurity may predict parental depression [19]. Thus, in order to examine associations between food insecurity and children’s mental health, it is important to control for individual and familial characteristics which may confound this association.

Past research linking food insecurity to children’s outcomes was mostly based on cross-sectional samples [5], [12], [14], [15] or short follow-up (up to two years) [16], [17] and the long-term consequences of exposure to food insecurity early on in life are not well known. In the present study, we test the relationship between food insecurity in early childhood (before age 4½) and children’s symptoms of depression/anxiety, aggression, and hyperactivity/inattention up to age 8, accounting for child and familial characteristics which may be associated with food insecurity and children’s mental health [16], [20]: child’s sex, immigrant status, family structure, maternal age at child’s birth, family income, maternal and paternal education, prenatal tobacco exposure, maternal and paternal depression, family functioning and negative parenting.

## Methods

Data for this study come from the Québec Longitudinal Study of Child Development (QLSCD) study, which follows a representative cohort of 2120 children born in the Canadian province of Québec in 1997–1998. To ensure geographic representation and minimize the effect of seasonality, participants were chosen through a random selection of children born throughout the year in each public health geographic area of the province. Twins and children with major diseases or handicaps at birth were excluded from the cohort. Selected children were first seen at 5 months of age and then once each year thereafter (follow-up assessments were conducted at 1½, 2½, 3½, 4½, 5, 6 and 8 years). Data on children and their parents were collected by trained interviewers through home interviews regularly conducted with the person most knowledgeable about the child (the mother in 98% of cases). Participating families gave written informed consent for the study at each assessment. The survey protocol was approved by the Quebec Institute of Statistics (Quebec City, Quebec, Canada) and the St-Justine Hospital Research Center (Montreal, Quebec, Canada) ethics committees. Informed consent for the study was obtained from parents or legal guardians.

**Table 1:** Characteristics of children and their families in relation to food insecurity: the Longitudinal Study of Child Development in Québec, 1997/98–2005.

	Food-insecure children n = 99	Non food-insecure children n = 1583	p-value
**Children’s characteristics**			
Sex (%):FemaleMale	46.553.5	50.549.5	0.44
Immigrant status (%):Non-immigrantImmigrant	80.819.2	88.311.7	**0.027**
Symptoms of depression/anxiety:LowIntermediateHigh	10.158.631.3	19.859.820.4	**0.0073**
Symptoms of aggression:LowIntermediateHigh	18.251.530.3	24.150.025.9	**0.35**
Symptoms of hyperactivity/inattention:LowLow/intermediateIntermediateHigh	17.237.430.315.2	21.138.335.15.4	**0.0011**
**Family characteristics**			
Family structure (%):Two-parent familyParents separated	49.550.5	77.222.8	**<0.0001**
Maternal age at child’s birth (%):> = 21 years<21 years	59.640.4	80.219.8	**<0.0001**
Family income (%):SufficientInsufficient	23.276.8	76.024.0	**<0.0001**
Maternal education (%):<High school degree> = High school degree	33.766.3	15.484.6	**<0.0001**
Paternal education (%):<High school degree> = High school degree	4654	18.481.6	**<0.0001**
Prenatal tobacco exposure (%):NoYes	62.637.4	75.924.1	**0.0030**
Maternal depression score (µ, se)	2.24, 1.52	1.28, 1.07	**<0.0001**
Paternal depression score (µ, se)	1.33, 1.10	0.97, 0.94	**0.0023**
Family functionning score (µ, se)	0.27, 0.17	0.25, 0.15	0.18
Negative parenting score (µ, se)	3.28, 1.18	2.98, 1.02	**0.0057**

**Table 2:** Food insecurity and children’s characteristics in relation to trajectories of psychological difficulties ages 4–8 years: the Longitudinal Study of Child Development in Québec, 1997/98–2005 (sex-adjusted ORs, 95% CI).

	High depression/anxietyprevalence: 21.0%OR (95% CI)	Highaggressionprevalence: 26.2%OR (95% CI)	Highhyperactivity/inattentionprevalence: 6.0%OR (95% CI)
Food insecurity :NoYes	11.79 (1.15–2.79)	11.21 (0.77–1.90)	13.06 (1.68–5.55)
Immigrant status:Non-immigrantImmigrant	10.90 (0.63–1.30)	10.91 (0.64–1.28)	10.89 (0.47–1.70)
Family structure:Two-parent familyParents separated	11.11 (0.85–1.45)	11.52 (1.19–1.95)	12.54 (1.67–3.85)
Maternal age at child’s birth:> = 21 years<21 years	10.93 (0.69–1.25)	11.55 (1.20–2.02)	12.33 (1.51–3.59)
Family income:SufficientInsufficient	11.28 (0.99–1.65)	11.40 (1.10–1.78)	12.11 (1.40–3.19)
Maternal education:> = High school degree< High school degree	11.08 (0.79–1.47]	11.42 (1.07–1.90)	11.80 (1.12–2.90)
Paternal education:> = High school degree< High school degree	11.08 (0.79–1.46)	11.80 (1.37–2.37)	12.87 (1.81–4.54)
Prenatal tobacco exposure:NoYes	10.88 (0.67–1.16)	11.60 (1.25–2.04)	11.96 (1.28–3.00)
Maternal depression score (per unit)	1.25 (1.14–1.38)	1.22 (1.11–1.34)	1.43 (1.24–1.64)
Paternal depression score (per unit)	1.11 (0.98–1.26)	1.12 (1.00–1.27)	1.26 (1.03–1.54)
Family functionning score (per unit)	1.70 (0.81–3.60)	2.68 (1.34–5.37)	2.86 (0.85–9.66)
Negative parenting score (per unit)	1.46 (1.30–1.63)	1.75 (1.56–1.96)	1.88 (1.57–2.26)

**Table 3:** Food insecurity and children’s trajectories of psychological difficulties ages 4–8 years: the Longitudinal Study of Child Development in Québec, 1997/98–2005 (multivariate ORs, 95% CI; beta, se)^1^.

	Highdepression/anxietyOR (95% CI)	HighaggressionOR (95% CI)	Highhyperactivity/inattentionOR (95% CI)
Food insecurity :NoYes	11.44 (0.78–2.66)	10.67 (0.35–1.29)	12.65 (1.16–6.06)
Sex:FemaleMale	10.80 (0.62–1.05)	12.07 (1.59–2.69)	12.46 (1.43–4.23)
Immigrant status:Non-immigrantImmigrant	10.65 (0.42–1.02)	10.79 (0.52–1.20)	10.69 (0.30–1.60)
Family structure:Two-parent familyParents separated	11.04 (0.72–1.50)	11.25 (0.89–1.77)	11.36 (0.74–2.49)
Maternal age at child’s birth:> = 21 years<21 years	10.94 (0.64–1.40)	11.25 (0.87–1.81)	11.55 (0.83–2.90)
Family income:SufficientInsufficient	11.05 (0.73–1.52)	11.05 (0.74–1.50)	10.90 (0.47–1.72)
Maternal education:> = High school degree< High school degree	11.25 (0.82–1.91)	10.98 (0.65–1.48)	10.77 (0.36–1.64)
Paternal education:> = High school degree< High school degree	10.88 (0.61–1.28)	11.51 (1.07–2.11)	11.80 (1.00–3.24)
Prenatal tobacco exposure:NoYes	10.68 (0.48–0.96)	11.18 (0.86–1.62)	11.11 (0.61–2.00)
Maternal depression score (per unit)	1.23 (1.08–1.39)	1.13 (1.00–1.28)	1.22 (0.99–1.51)
Paternal depression score (per unit)	1.03 (0.89–1.18)	1.03 (0.90–1.19)	1.12 (0.88–1.43)
Family functionning score (per unit)	1.93 (0.77–4.81)	2.68 (1.13–6.37)	1.15 (0.23–5.87)
Negative parenting score (per unit)	1.42 (1.24–1.62)	1.70 (1.49–1.93)	1.75 (1.39–2.20)

1 The ORs presented are adjusted for all the variables in each column.

The average response rate during the 8 years of data collection was 87.0% (range, 68%–100%) [20]. The present analysis is based on 1682 children with available data on food insecurity as well as at least 2 measures of mental health symptoms. Compared to the original cohort, nonparticipants were more likely to be from families that were characterized by low income, low education, immigrant background, young maternal age, single-parenthood and maternal depression, but participants and nonparticipants did not differ with regard to children’s mental health symptoms.

### Food Insecurity

Food insecurity was ascertained when the participating child was 1½ and 4½ years old. On those two occasions, mothers were asked: a) whether family members had eaten less than they should have because they had run out of food or money to buy food (1½ and 4½ years), b) whether family members had eaten the same foods several times because they did not have anything else and could not afford to buy other foods (4½ years only), c) whether the family could not afford to offer nutritious meals to the children (4½ years only), d) how often family members did not eat as much as they should have because they had run out of food or money to buy food (4½ years). These measures of food insecurity were previously shown to predict children’s overweight and obesity [21], [22]. Children whose families experienced any of these situations were considered to be exposed to food insecurity: (3.4% of the study population at age 1½ year, 3.6% at age 4½ years, 5.9% at 1½ or 4½ years of age).

### Children’s Mental Health

Children’s mental health was assessed at 4½, 5, 6 and 8 years based on parental reports. Symptoms of depression/anxiety were assessed using 5 items adapted from the Preschool Behavior Questionnaire [23] and the Child Behavioral Checklist [24]: ‘nervous, high strung or tense’, ‘fearful or anxious’, ‘worried’, ‘not as happy as other children’, ‘has difficulty having fun’ [25]. Symptoms of aggression were assessed using 5 items previously validated in this study: ‘hits’, ‘kicks’, ‘bites’, ‘fights’, ‘bullies others’ [26]. Symptoms of hyperactivity-impulsivity and inattention were assessed through a combination of items from the Child Behavior Checklist [24], the Ontario Child Health Study Scales [27] and the Preschool Behavior Questionnaire [28]. Hyperactivity-impulsivity was assessed using 5 items: ‘can’t sit still, is restless’, ‘fidgets’, ‘can’t settle down to do anything for more than a few moments’, ‘is impulsive, acts without thinking’, ‘has difficulty waiting for turn in games’ [29]. Inattention was assessed using 3 items: “can't concentrate, can't pay attention for long”, “is easily distracted, has trouble sticking to any activity”, “is inattentive”. All items pertaining to children’s mental health symptoms were scored 0 (‘never’), 1 (‘sometimes’) or 2 (‘often’) and then summed to range 0–10 [20].

Based on the four measures of children’s psychological symptoms between ages 4½ and 8 years which were available to us, we used semiparametric mixture models [30] to calculate longitudinal symptom trajectories. This approach makes it possible to identify groups with distinct longitudinal symptom patterns empirically rather than using a set cut off. As such, this method provides a description of the ‘natural’ course of the evolution of mental health symptoms over time. Additionally, the reliance on multiple measures of symptoms as well as the grouping of children according to a trajectory pattern reduces the measurement in error related to a single assessment [31]. For each symptom group, the model implemented using the PROC TRAJ procedure in SAS defined the shape of the trajectory and the proportion of participants in each group. The validity of the ‘best fitting” classification was confirmed using the Bayesian Information Criterion (BIC). Overall, we identified 3 groups of symptoms of depression/anxiety (low: 19.2%, moderate: 59.8%, high: 21.0%), 3 groups of symptoms of aggression (low: 23.8%, moderate/declining: 50.1%, high: 26.2%), and 4 groups of symptoms of hyperactivity/inattention, (low: 20.9%, low/intermediate: 38.3%, intermediate: 34.8%, high: 6.0%). Children’s symptoms were moderately correlated to one another (correlation coefficients at age 8 years: depression/anxiety and aggression: 0.16, p<0.0001; depression/anxiety and hyperactivity/inattention: 0.31, p<0.0001; aggression and hyperactivity/inattention: 0.32, p<0.0001).

### Covariates

Analyses were adjusted for the characteristics of children and their families, which can be associated with food insecurity and children’s mental health symptoms [16]. Covariates were measured at age 5 months (prenatal tobacco exposure, maternal and paternal depressive symptoms and family functioning) or concomitantly to food insecurity. Demographics included the child’s sex (male vs. female), immigrant status (immigrant vs. non-immigrant), family structure (parents separated vs. two-parent family) and maternal age at child’s birth (<21 vs. > = 21 years). Family income was calculated according to guidelines issued by Statistics Canada, taking into account the number of people in the household and the type of residence area (urban vs. rural based on population density); family income was coded as insufficient vs. sufficient. Maternal and paternal education was defined as <High school vs. > = High school. Prenatal tobacco exposure was defined as maternal consumption of > = 1 cigarette/day (yes vs. no). Maternal and paternal depressive symptoms were assessed by the abbreviated version (12 items) of the Center for Epidemiologic.

Studies Depression (CESD) Scale [32]. Parents reported the frequency of depressive symptoms in the previous week. Each item was coded on a 4-point scale. Total informant ratings were z-standardized. Family dysfunction was assessed with the McMaster Family Assessment, which includes 12 items measuring communication, showing and receiving affection, control of disruptive behaviour, and problem resolution in the family; each item was coded 0 (‘never’), 1 (‘sometimes’), or 2 (‘often’) and the overall score was *z*-standardized [33]. Negative parenting was assessed using the Parental Cognition and Conduct Toward the Infant Scale, which includes dimensions such as coercitive parenting (7 items) and overprotection (5 items), each rated on a scale ranging 0 to 10; overall scores were z-standardized [34].

### Statistical Analysis

To study the association between food insecurity and children’s mental health outcomes, we combined exposure to food insecurity when children were 1½ and 4½ years of age (ever food-insecure vs. never food-insecure) and tested associations with children’s probability of being on a ‘high’ behavioural trajectory group at ages 4½ to 8 years. First, we tested sex-adjusted associations, in order to account for sex-related differences in the prevalence of mental health symptoms in children. Second, we adjusted for covariates. In additional analyses we tested whether the association between food insecurity and long-term behavioural problems 1) was robust to statistical adjustment on behavioural problems prior to age 4½; 2) differed depending on the child’s sex. Analyses were carried out in a logistic regression framework in SAS (V9).

## Results

5.9% of study children experienced food insecurity between ages 1½ and 4½ years. As shown in **Table 1**, food insecurity was associated with characteristics of children and their families, including immigrant status, family structure, maternal age at child’s birth, family income, maternal and paternal education, prenatal tobacco exposure, maternal and paternal depression and negative parenting. In sex-adjusted regression analyses (**Table 2**), compared to unexposed children, children who experienced food insecurity were more likely to have persistently high levels of symptoms of depression/anxiety (OR: 1.79, 95% CI 1.15–2.79) and hyperactivity/inattention (OR: 3.06, 95% CI 1.68–5.55), but not aggression. In multivariate regression models adjusted for characteristics of children and their families (**Table 3**), the association between food insecurity and symptoms of depression/anxiety decreased and became statistically non-significant (fully adjusted OR: 1.44, 95% CI 0.78–2.66); however the association between food insecurity and symptoms of hyperactivity/inattention remained elevated and statistically significant (fully adjusted OR: 2.65, 95% CI 1.16–6.06). The decrease in ORs associated with food insecurity was greatest after controlling for maternal depression (41% for symptoms of depression/anxiety and 42% for symptoms of hyperactivity/inattention). In additional analyses further adjusted for hyperactivity/inattention at age 1½ years, the association between food insecurity and hyperactivity/inattention between ages 4½ and 8 years remained elevated but lost statistical significance (OR: 2.18, 95% CI 0.60–8.00). Sex-stratified analyses showed no significant differences in the association between food insecurity and hyperactivity/inattention in boys and girls and the interaction test was not statistically significant (p = 0.88).

## Discussion

In a birth cohort study of families with young children followed for up to 8 years, we found that food insecurity predicted children’s two-fold increase in the likelihood of persistent hyperactivity/inattention, even after accounting for family socioeconomic circumstances and parental mental health, although this association lost statistical significance when further adjusted for children’s behavioural symptoms at age 1½ years. To our knowledge, this is the first study to examine the relationship between food insecurity and children’s mental health over such an extended follow-up, independently of individual and family characteristics known to predict children’s outcomes. Our finding contributes to growing scientific evidence of the impact of food insecurity on children’s well-being, and suggests that exposure very early in life can have lasting effects on development.

### Limitations and Strengths

Prior to discussing the study findings, we need to acknowledge methodological limitations: 1) due to selective attrition which often occurs in longitudinal cohort studies, the study sample included fewer children from socioeconomically disadvantaged families than the original cohort; thus, the prevalence of food insecurity among Canadian families with small children may be higher than we report; 2) children were not assessed for clinically significant emotional and behavioural problems, barring conclusions regarding the impact of food insecurity on psychological problems that require medical attention; nevertheless, children who experience mental health difficulties early on are at risk of psychiatric disorders later in life implying that symptoms such as the ones we measured require attention from parents, teachers and physicians [35]; 3) children’s mental health symptoms were assessed by their mothers, raising the possibility of reporting bias, particularly if the mother was depressed; evidence that maternal reports of children’s behaviour coincide with reports of other individuals in children’s environment (father, other family, friends) [25], [36], [37] implies that symptoms picked up by mothers are valid; nevertheless future investigations should account for teacher ratings, particularly to measure hyperactivity/inattention, as a way to alleviate problems related to potential shared method variance; 4) food insecurity was measured using four items, which may have led us to underestimate its occurrence as not all aspects of food shortage and inadequacy were assessed; additionally, we did not have sufficient statistical power to study children’s mental health in relation to changes in food insecurity over time.

Our study also has key strengths: 1) analyses were based on a community sample and we were able to estimate the burden of behavioural problems associated with food insecurity among children in the general population, while most prior studies focused on high-risk families; 2) longitudinal follow-up of children’s mental health allowed us to distinguish different types of symptoms and their developmental patterns over up to 7 years of follow-up; 3) statistical adjustment for multiple individual and family factors potentially associated with children’s outcomes.

### Food Insecurity and Children’s Behaviour

Our finding of an association between exposure to food insecurity and children’s mental health symptoms is consistent with prior research conducted cross-sectionally or over a limited follow-up [5], [12], [38]. In contrast to studies conducted in high-risk [12], [14] or older samples [15], [16], the association between food insecurity and children’s symptoms of emotional problems in our study disappeared after we adjusted for individual and family factors.

Adding to prior research which did not always distinguish specific aspects of children’s behaviour, we found that food insecurity is distinctively associated with children’s symptoms of hyperactivity/inattention. This association lost statistical significance after adjusting for children’s behavioural difficulties at age 1½ years, but did not much change, which may be due to the small number of cases of hyperactivity/inattention in our study and calls for additional research in larger samples.

The association between food insecurity and children’s behaviour may reflect several mechanisms. First, food insecure families are disproportionately exposed to multiple risks which can impair children’s development and mental health, including poverty, marital discord, single parenthood, violence, parental substance abuse and psychopathogy [39], [40]. Our analyses are controlled for income, family structure and functioning, as well as parental psychopathology and attitude towards children, but we cannot entirely rule out the possibility of residual confounding, whereby the association between food insecurity and children’s behaviour is not causal but rather reflects the co-occurrence of other risk factors. Second, through psychological pathways, food insecurity early in life may lead to weak attachment between parents and children, which can have negative consequences on children’s mental health later on [41]. Third, food insecurity may be associated with maternal depression, which, in turn, impacts on child mental health [19]. Fourth, food insecurity may directly predict the occurrence of behavioural difficulties through inadequate nutrition [18]. In particular, compared to non food-insecure children, food-insecure children’s diets are high in fat, refined sugars and sodium and low in fruits, vegetables and fiber [42], leading to high carbohydrate intake [43] and decreased levels of vitamin, omega-3, fatty acids and iron [44], [45]. High consumption of refined sugars as well as iron-deficiency anaemia may have behavioural consequences such as hyperkinesia, inattention and poor memory, and may contribute to the link between food insecurity and hyperactivity/inattention. Although there is controversy regarding the importance of dietary factors in relation to ADHD risk, recent evidence from intervention trials suggests that the introduction of a healthy diet yields improvements in symptoms in some children [46], [47]. Given the burden of ADHD in children and adults [48], and the impact of hyperactivity/inattention on children’s concurrent and future health, academic, and social outcomes [49]–[51], ensuring children’s mental health should be a public health priority.

### Conclusions

Children growing up in food-insecure families are two-times more likely to have high levels of persistent symptoms of hyperactivity/inattention than children who are not food insecure. Reducing the burden of food insecurity in families could help decrease the burden of mental health problems in school-aged children and reduce social inequalities in development.
